# Impact of frailty, biomarkers and basic biochemical parameters on outcomes of comatose patients in status epilepticus: a single-center prospective pilot study

**DOI:** 10.1186/s12883-024-03537-y

**Published:** 2024-01-26

**Authors:** Zdenek Krejzar, David Sila, Petr Waldauf, Eduard Kuriscak, Petr Mokrejs, Vera Spatenkova

**Affiliations:** 1 Department of Neurology, First Faculty of Medicine, Charles University in Prague, Katerinska 1660/32, Prague 2, 121 08 Czech Republic; 2 Centrum of Anaesthesiology, Resuscitation and Intensive Care, Regional Hospital, Husova 357/10, Liberec, 46001 Czech Republic; 3 Emergency Medical Services, Klasterni 954/5, Liberec, 460 01 Czech Republic; 4 Faculty of Health Studies, Technical University in Liberec, Studentska 1402/2, 461 17, Liberec 1, Czech Republic; 5 Department of Anaesthesiology and Resuscitation, Third Faculty of Medicine, Charles University in Prague, Ruska 10, Prague, 100 00 Czechia; 6 University Hospital Kralovske Vinohrady, 110 34 Prague 10, Srobarova, 1050 Czech Republic; 7 Institute of Physiology, First Faculty of Medicine, Charles University in Prague, Albertov 5, Prague, 128 00 Czech Republic; 8 Neurocenter, Neurointensive Care Unit, Regional Hospital, Husova 357/10, Liberec, 46001 Czech Republic

**Keywords:** Status epilepticus, Frailty, Score, Outcome, STESS, CFS, mFI-11

## Abstract

**Background:**

Status epilepticus (SE) is a severe acute condition in neurocritical care with high mortality. Searching for risk factors affecting the prognosis in SE remains a significant issue. The primary study’s aim was to test the predictive values of the Clinical Frailty Scale (CFS) and the Modified 11-item Frailty Index (mFI-11), the biomarkers and basic biochemical parameters collected at ICU on the Glasgow Outcome Scale (GOS) assessed at hospital discharge (hosp), and three months later (3 M), in comatose patients with SE. The secondary aim was to focus on the association between the patient’s state at admission and the duration of mechanical ventilation, the ICU, and hospital stay.

**Methods:**

In two years single-centre prospective pilot study enrolling 30 adult neurocritical care patients with SE classified as Convulsive SE, A.1 category according to the International League Against Epilepsy (ILAE) Task Force without an-/hypoxic encephalopathy, we evaluated predictive powers of CFS, mFI-11, admission Status Epilepticus Severity Score (STESS), serum protein S100, serum Troponin T and basic biochemical parameters on prognosticating GOS using univariate linear regression, logistic regression and Receiver Operating Characteristic (ROC) analysis.

**Results:**

Our study included 60% males, with a mean age of 57 ± 16 years (44–68) and a mean BMI of 27 ± 5.6. We found CFS, mFI-11, STESS, and age statistically associated with GOS at hospital discharge and three months later. Among the biomarkers, serum troponin T level affected GOS hosp (*p* = 0.027). Serum C-reactive protein significance in prognosticating GOS was found by logistic regression (hosp *p* = 0.008; 3 M *p* = 0.004), and serum calcium by linear regression (hosp *p* = 0.028; 3 M *p* = 0.015). In relation to secondary outcomes, we found associations between the length of hospital stay and each of the following: age (*p* = 0.03), STESS (*p* = 0.009), and serum troponin T (*p* = 0.029) parameters.

**Conclusions:**

This pilot study found promising predictive powers of two frailty scores, namely CFS and mFI-11, which were comparable to age and STESS predictors regarding the GOS at hospital discharge and three months later in ICU patients with SE. Among biomarkers and biochemical parameters, only serum troponin T level affected GOS at hospital discharge.

**Supplementary Information:**

The online version contains supplementary material available at 10.1186/s12883-024-03537-y.

## Background

Status epilepticus (SE) is a frequent and severe acute condition in neurocritical care with a high mortality rate [1]. Early prognosis assessment can significantly influence the management of SE cases [2]. However, relevant risk factors correlating with poor outcomes in SE have not been well established yet [3]. Most relevant clinical risk factors include age, level of consciousness before treatment, type of seizures (worst seizure type, history of seizures), comorbidities (and complications of SE), etiology of SE (acute, progressive, unknown) and history of epilepsy, critically ill patients (severely ill patients in intensive care units) and refractory cases [4], barbiturates and numbers of used antiseizure medications, and insufficient adherence to medication [5–9]. Currently, these factors form nine risk models - prognostic scores of SE used to date, with variable prognostic performance depending on the heterogeneity of evaluated patient cohorts [10].

A biochemical examination is a routine procedure during admission to intensive care. Among biochemical markers, routine laboratory blood parameters (such as potassium, sodium, chloride, creatinine, urea, bilirubin, platelet and white blood cell count, etc.), as well as brain injury biomarkers found in blood or cerebrospinal fluid, e.g., the neuron-specific enolase, S100-beta protein, progranulin, could be mentioned [11]. Likewise, despite recent progress in establishing specific associations between evaluated biomarkers and SE prognosis, more precise quantification of the prognostic power of available biomarkers and essential biochemical parameters needs to be established [12, 13].

Frailty has been recognized as a risk factor for many medical and surgical conditions, including critical illness from various causes [14–17]. However, the quantification of frailty impact on SE outcomes still needs to be elaborated more thoroughly. The primary aim of this study was to test the predictive power of ICU admission frailty scores CFS and mFI-11, and the biomarkers and basic biochemical parameters, on outcomes of comatose patients with SE. The analysed outcome was the Glasgow Outcome Scale (GOS) obtained at hospital discharge (GOS hosp) and three months later (GOS 3 M). The secondary aim of our study was to focus on associations between patients´state at admission and the duration of mechanical ventilation, the ICU, and hospital stay.

## Materials and methods

We conducted a 2-year single-center prospective pilot study in a 10-bed general ICU that serves our neurocritical care patients.

We included all consecutive patients who fulfilled the entry criteria: (1) adult patients with age ≥ 18 years; (2) SE classified as Convulsive SE, A.I category, according to the International League Against Epilepsy (ILAE) Task Force [18]; (3) admitted to our ICU between 1st January 2018 and 31st December 2019. Our exclusion criteria were: (1) patients younger < 18 years, (2) an-/hypoxic encephalopathy (all patients with cardiac arrest and successful cardiopulmonary resuscitation were excluded).

### Characteristics of the study population

We evaluated the following characteristics and demographic data: age, weight, height, body mass index (BMI), and gender. We also monitored the presence of alcohol intoxication at admission, alcohol and nicotine abuse in medical history, duration of mechanical ventilation, ICU length of stay (LOS), and hospital LOS. Detailed characteristics of our cohort and collected parameters are in Tables 1 and 2.

### Scores

We calculated the following scores of each patient: (1) Glasgow Coma Scale (GCS onset, it was assessed by the emergency medical services doctor at the intervention site before administering sedative medication and intubation) - upon admission to the ICU, patients were already under the influence of analgosedation; (2) Status Epilepticus Severity Score at admission (STESS) [2]. We evaluated frailty levels prior to each patient´s onset of an acute illness using two validated scores: (3) Clinical Frailty Scale (CFS) [19]; (4) Modified 11-item Frailty Index (mFI-11) [19]. The required information was obtained from an ambulance, family, caregivers, or hospital records. We also measured the (5) Glasgow Outcome Scale: five-degree scale (1- Death; 2 – Neurovegetative state; 3 – Severe disability; 4 – Moderate disability; 5 - Good recovery) at hospital discharge (GOS hosp) and (6) Glasgow Outcome Scale assessed three months later (GOS 3 M). The favorable outcome was defined as GOS 4–5.

### Biomarkers and biochemical parameters

We assessed the following serum biomarkers at admission: (1) Protein S100 (S100) and (2) high-sensitivity Troponin T(TNT). These parameters were measured by electrochemical luminescence on the Cobas Pro system (Roche Diagnostics, Basel, Switzerland). In addition, we tested the following basic biochemical parameters: (1) serum glucose (Gly); (2) serum natrium (Na); (3) serum potassium (K); (4) serum chloride (Cl); (5) serum magnesium (Mg); (6) serum phosphorus (P); (7) serum calcium (Ca); (8) serum albumin (Alb); (9) serum osmolality (Sosm); (10) osmotic gap (OG); 11) serum C-reactive protein (CRP); 12) blood pH; 13) blood lactate (Lac); 14) blood base excess (BE). The following biochemical parameters were measured on the Cobas Integra 800 system (Roche Diagnostics, Basel, Switzerland): Na, K, and Cl by direct/indirect potentiometry and Mg, P, Ca, and Alb using photometry. The cryoscopic osmometer Osmo Station (Akray, Inc., Japan) was used to measure Sosm. Arterial blood pH, Lac, and BE were measured on the blood gas analyzer ABL 800 (Radiometer, Copenhagen, Denmark).

### Statistical analysis

The analyses were performed using the statistical software R version 4.2.21 [20]. Exploratory data analysis was done for all parameters. Continuous parameters are reported as mean ± SD or median and IQR (interquartile range) according to the normality of the distribution analyzed by the Shapiro-Wilk test and categorical parameters as counts and percentages. Continuous parameters were compared using a t-test or Wilcoxon test, and categorical parameters using chi-square or Fisher exact test as appropriate. Continuous explanatory parameters were analyzed in two forms: (1) as they were – untransformed, and (2) processed by Yeo-Johnson transformation and then standardized. Univariate linear regression analysis was performed to evaluate the association between explanatory variables and GOS. To predict GOS into favourable (GOS 4–5) and unfavorable (GOS 1–3) conditions, the univariate logistic regression was modelled using binarized explanatory variables dichotomized by optimized cut-off points obtained by maximization of the Youden index [21]. Receiver Operating Characteristic (ROC) analysis was performed on explanatory variables to optimize the discrimination accuracy of tested GOS estimators. *P*-values < 0.05 were considered statistically significant for our tests and estimators.

## Results

During the 2-year study period, we enrolled 30 patients. The baseline characteristics of the study population can be seen in Table 1, and the overview of basic biochemical parameters in Table 2. Detailed descriptive characteristics of dependent and independent (explanatory) variables are depicted in Suppl. Tables 1, and for better illustration as boxplots in Suppl. Figure 1, along with the correlation coefficients between measured variables in Suppl. Figure 2.

Favorable outcome (GOS 4–5) at discharge from the hospital was achieved in 17 patients (56,6%) and 15 patients (50%) three months later. The median duration of mechanical ventilation was 52 (IQR 22–202) hours, median ICU LOS 5 (IQR 2–11) days, and median hospital LOS 14 (8–26) days.

**Fig. 1:**
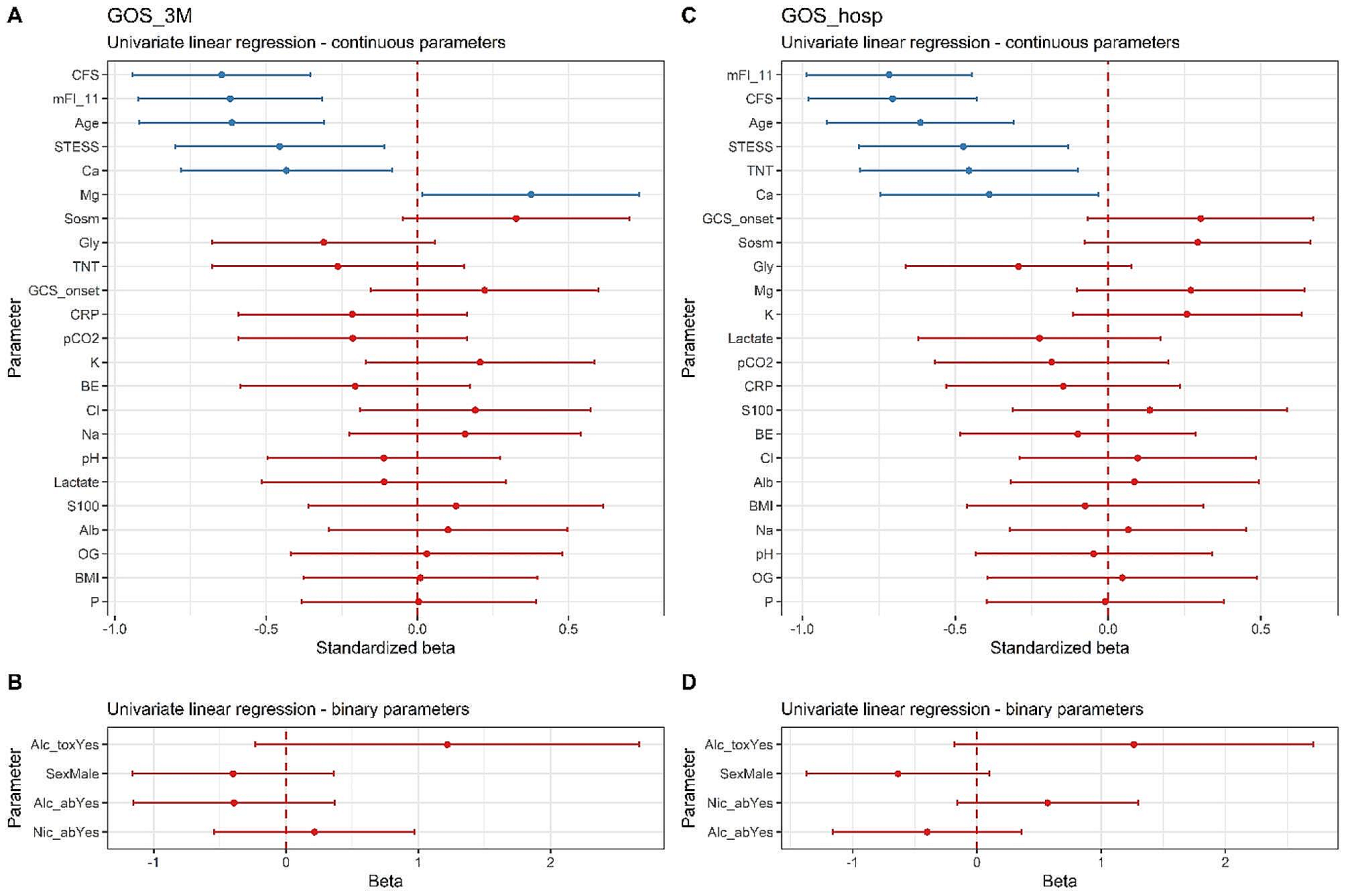
Forest plots showing the coefficients of linear regression and their respective confidence intervals (CI) indicating predictive powers of the analyzed explanatory variable on GOS (evaluated after the Yeo-Johnson transformation and standardization). Plots A and C show the values of standardized beta for continuous explanatory parameters, and plots B and D show the beta for categorical explanatory variables. The higher the beta, the higher the predictive power of the given explanatory variable (with negative values meaning negative correlations and vice versa). Parameters whose 95% CI included zero (95% CI equals the depicted bar width) did not affect GOS in a statistically significant way (*p* > 0.05, in red) Conversely, parameters in blue were identified as relevant predictors regarding our dataset (*p* < 0.05) and evaluated by linear regression. GOS hosp: Glasgow Outcome Scale at hospital discharge; GCS 3 M: Glasgow Outcome Scale three months after hospital discharge; STESS: Status Epilepticus Severity Score at admission; CFS: Clinical Frailty Scale; mFI-11: Modified 11-item Frailty index; GCS: Glasgow Coma Scale; BMI: body mass index; Alc_ab: alcohol abuse; Alc_tox: alcohol intoxication; Nic_ab: nicotine abuse; S100: serum protein S-100; TNT: serum high-sensitivity Troponin T; Na: serum natrium; K: serum potassium; Cl: serum chloride; Mg: serum magnesium; P: serum phosphorus; Ca: serum calcium; Alb: serum albumin; CRP: C-reactive protein; Lac: lactate; BE: base excess; Sosm: serum osmolality; OG: osmotic gap

In Fig. 1, the forest graphs illustrate the linear regression coefficients, which indicate the strength of association between explanatory parameters (after transformation and standardization) and GOS outcomes. The graphs indicate which parameters could be considered significant in predicting GOS hosp and GOS 3 M regarding our cohort. In Suppl. Tables 2a and 2b, the detailed list of all parameters of the estimated linear regression model for GOS hosp and GOS 3 M, can be seen. Based on the described univariate linear regression model, the following statistically significant predictive factors were identified: CFS, mFI-11, AGE, STESS, and Ca for both GOS hosp and GOS 3 M, with Mg significantly affecting GOS 3 M only.

**Fig. 2:**
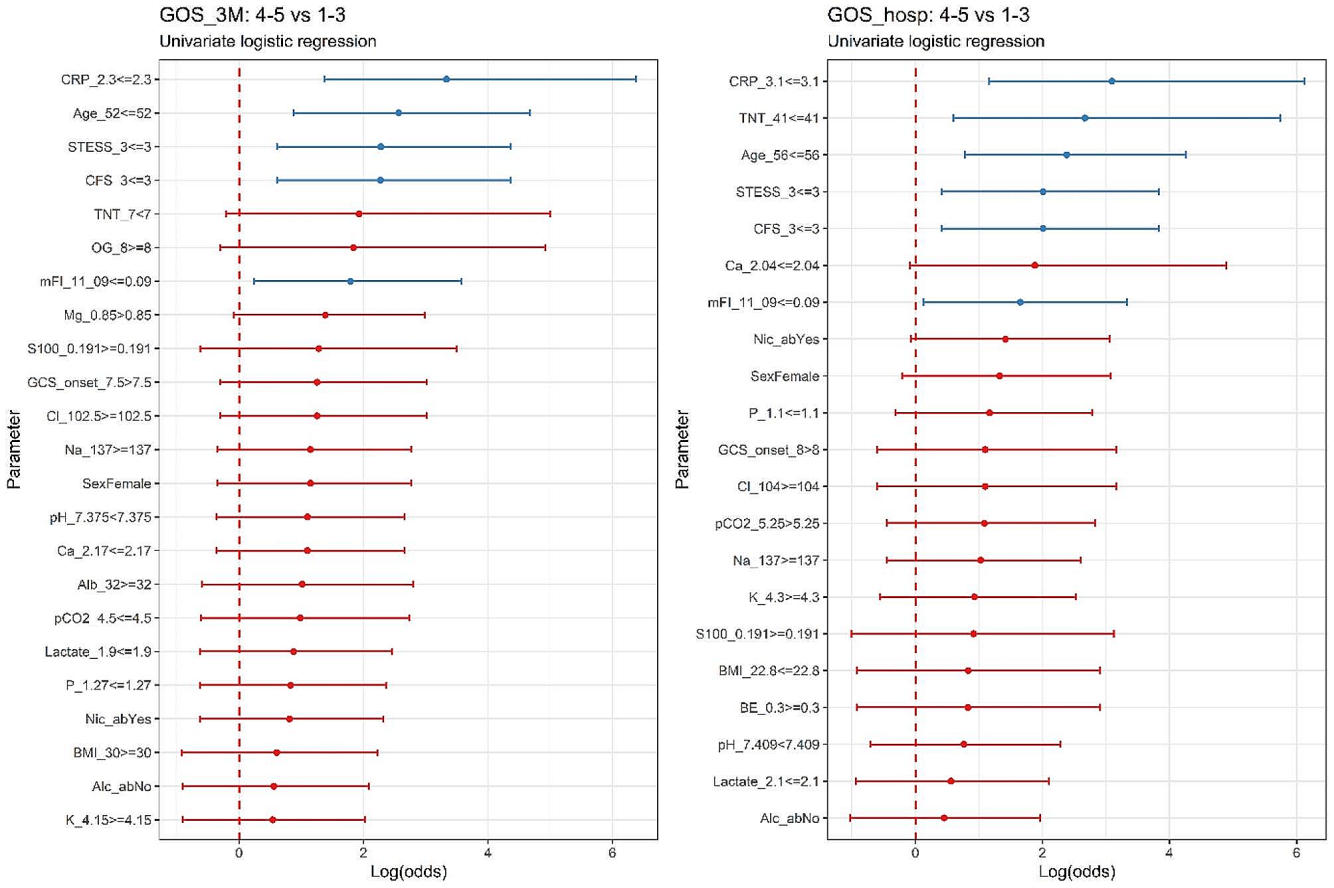
Forest plots showing the characteristics of the logistic regression model indicating the predictive powers of respective logistic classifiers (characterized mainly by odds ratios - OR and its 95% confidence intervals - CI), estimating the GOS from explanatory binarized variables dichotomized by optimal cut-off points. On the ‘log(odds)’ or logit axis, the depicted intervals correspond to ln (95% CI) of estimated OR listed in Suppl. Table 2 with middle bold dots matching the ln (OR). OR represents the ratio of ‘odds of becoming GOS 4–5 if the predictor parameter < than the cut-off value’ / ‘odds of becoming GOS 4–5 if predictor parameter is > than the cut-off value. The further the OR is from zero, the higher the predictive power of the corresponding binarized explanatory variable. Variables in blue are considered statistically significant (*p <* 0.05), representing the identified predictors regarding our dataset analyzed by logistic regression. OR: odds ratio; CI: confidence interval; GOS hosp: Glasgow Outcome Scale at hospital discharge; GCS 3 M: Glasgow Outcome Scale three months after hospital discharge; STESS: Status Epilepticus Severity Score at admission; CFS: Clinical Frailty Scale; mFI-11: Modified 11-item Frailty index; GCS: Glasgow Coma Scale; BMI: body mass index; Alc_ab: alcohol abuse; Alc_tox: alcohol intoxication; Nic_ab: nicotine abuse; S100: serum protein S-100; TNT: serum high-sensitivity Troponin T; Na: serum natrium; K: serum potassium; Cl: serum chloride; Mg: serum magnesium; P: serum phosphorus; Ca: serum calcium; Alb: serum albumin; CRP: C-reactive protein; Lac: lactate; BE: base excess; Sosm: serum osmolality; OG: osmotic gap

In Fig. 2, the results quantifying the predictive value of binarized exploratory variables are shown, demonstrating estimated odds ratios (OR) and their respective 95% CI, indicating the strength of association between binarized explanatory variables (split using optimized cut-off points) and two valued GOS (1–3 vs. 4–5), determining the plausibility of respective logistic estimators regarding our dataset. In Suppl. Table 3, a detailed list of corresponding parameters of the used logistic regression model could be compared for GOS hosp and GOS 3 M.

In addition to the ORs and CIs shown in Fig. 2, which outline the predictive values of tested variables, the explorative data analysis can be found in Suppl. Table 4. It comprehensibly illustrates the predictive values of the most significant predictors after their binarization. Suppl. Figure 3 illustrates the same aspect visually for the most relevant GOS predictors identified in our data. The binarization was done based on the maximization of the Youden index, which helped us find optimal cut-off points of respective logistic regression GOS classifiers. ROC analysis of the most significant predictor parameters used to obtain the respective cut-off points can be seen in Suppl. Figure 4.

Comparing the predictive powers of two frailty scores, CFS and mFI-11, and the predictive powers of age and STESS on GOS, it can be concluded that they had comparable associations with GOS measured at hospital discharge and three months later. We did not find much significant associations between the explanatory variables and the secondary outcomes defined as the duration of mechanical ventilation, the ICU, and the hospital stay. However, a dependence was identified between hospital stay and the age (*p* = 0.03), the STESS (*p* = 0.009) and the serum troponin T (*p* = 0.029), as indicated by the parameters of standardized model of linear regression.

**Table 1:** Baseline characteristics of our cohort and scores we analyzed

Parameter	Unit	N (%) Mean ± SD
Gender male		18 (60%)
Age	years	56.53 ± 15.9
Body mass index	kg/m^2^	27.07 ± 5.57
Height	cm	168.57 ± 7.78
Weight	kg	77.27 ± 18.63
Alcohol abuse		12/18 (40.0%)
Alcohol intoxication		2/28 (6.7%)
Nicotine abuse		15/15 (50%)
CFS 1 2 3 4 6 7 8		30 3.0 (10.0%) 6.0 (20.0%) 10.0 (33.3%) 6.0 (20.0%) 2.0 (6.7%) 2.0 (6.7%) 1.0 (3.3%)
mFI-11 0 8 0.09 0.1 0.27 0.36 0.45 0.54 0.72		30 8.0 (26.7%) 10.0 (33.3%) 1.0 (3.3%) 8 2.0 (6.7%) 4.0 (13.3%) 3.0 (10.0%) 1.0 (3.3%) 1.0 (3.3%)
GCS onset		
3 5 6 7 8 9 10 11 13		8.0 (26.7%) 1.0 (3.3%) 7.0 (23.3%) 4.0 (13.3%) 2.0 (6.7%) 4.0 (13.3%) 2.0 (6.7%) 1.0 (3.3%) 1.0 (3.3%)
STESS at admission		
2 3 4 5 6		11.0 (36.7%) 8.0 (26.7%) 4.0 (13.3%) 6.0 (20.0%) 1.0 (3.3%)
GOS hospital discharge 1 2 3 4 5 GOS 3 month 1 2 3 4 5		30 4 (13.3%) 1 (3.3%) 8 (26.7%) 7 (23.3%) 10 (33.3%) 30 10 (33.3%) 1 (3.3%) 4 (13.3%) 5 (16.7%) 10 (33.3%)

SD: standard deviation; GCS: Glasgow Coma Scale; STESS: Status Epilepticus Severity Score; CFS: Clinical Frailty Scale; mFI-11: Modified 11-item Frailty Index; GOS: Glasgow Outcome Scale.

**Table 2:** Biomarkers and basic biochemical parameters estimated at admission

Parameter	Unit	Mean ± SD Median (IQR)
S100 TNT Gly Na K Cl Mg P Ca CRP Alb pH pCO_2_ BE Lac Sosm OG	ug/l µmol/l mmol/l mmol/l mmol/l mmol/l mmol/l mmol/l mmol/l mg/l g/l kPa mmol/l mmol/l mmol/kg mmol/kg	0.14 (0.10–0.26) 20 (9.0–56) 7.45 (6.60–8.67) 135.27 ± 6.55 4.11 ± 0.64 98.03 ± 8.10 0.88 ± 0.16 1.20 (0.99–1.43) 2.20 ± 0.19 5.0 (1.0–18.0) 34.57 ± 4.88 7.36 ± 0.10 4.98 (4.41–5.67) -3.2 (-6.2–0.0) 2.00 (1.12–3.38) 288.04 ± 16.82 1.0 (-4.0–6.0)

SD: standard deviation; IQR: interquartile range; S100: serum protein S-100; TNT: serum high-sensitivity Troponin T; Na: serum natrium; K: serum potassium; Cl: serum chloride; Mg: serum magnesium; P: serum phosphorus; Ca: serum calcium; Alb: serum albumin; CRP: C-reactive protein; Lac: lactate; BE: base excess; Sosm: serum osmolality; OG: osmotic gap.

## Discussion

Status epilepticus is a common and severe emergency in neurology with high mortality rates of up to 38% [1]. Currently, SE-specific scores like Status Epilepticus Severity Score (STESS), Epidemiology-based Mortality score in Status Epilepticus (EMSE), and modified STESS (mSTESS) are available to predict outcomes in SE patients [3]. However, their prognostic value, especially in patients who require intensive care, is limited [4]. In addition, many researchers see their weakness in that these scores do not consider organ dysfunction and physiological reserve beyond the brain. For this reason, they tested the predictive value of complex illness severity scoring systems, like Simplified Acute Physiology Score II (SAPS II), Acute Physiology and Chronic Health Evaluation II (APACHE II), and Sequential Organ Failure Assessment (SOFA) Score in SE. Although their performance in ICU patients was better than SE-specific scores, they still need more predictive power to be used separately on their own [4, 22].

Therefore, we looked at the topic from a new perspective. Frailty poses a biological syndrome of decreased physiological reserves that result in diminished resiliency, loss of adaptive capacity, and increased vulnerability to stressors [23]. For that reason, it might offer the missing level of prognostic information to the standard prognostic scores in SE. All previously mentioned SE-specific scores include age as their essential component. However, age and aging are not the same, and frailty is the reason why. Unsurprisingly, previous research proved the impact of frailty on outcomes in many medical and surgical conditions, including critical illness of different etiologies [14–17]. As far as we know, our study is the first one evaluating and quantifying the prognostic value of frailty of SE patients in the ICU.

There are several validated tools to screen for and quantify frailty. We chose two well-known ones to evaluate our patients. The first one is the CFS, and the second is the mFI-11. The CFS is an intuitive tool based on the pictographic description and information that defines nine classes from very fit to terminally ill patients [24]. The mFI-11 is an index developed by Velanovich et al. by condensing the original frailty index of 92 items into 11 variables [17, 19]. The mFI-11 reflects the patient’s medical history and activity of daily living and ranges gradually from 0 to 1, e.g. in the case of 3 positively screened items, the mFI-11 = 3/11 = 0.27. To achieve a good homogeneity of the studied cohort, we focused on a subgroup of comatose patients with status epilepticus and prominent motor symptoms classified as A.1 category (Convulsive SE) according to the Classification of SE proposed by the ILAE Task Force [18].

Both frailty scores successfully predicted our study’s outcomes at hospital discharge and outcomes three months later rated by GOS. Although they were not superior to age alone or STESS score, they have shown comparable predictive power measured by linear or logistic regression. The strength of the association of relevant explanatory variables with GOS slightly differed depending on whether it was assessed from linear- or logistic regression-based models, as seen by comparing Figs. 1 and 2. The difference was caused mainly by the binarization of explanatory variables in logistic regression, dichotomizing them using optimized cut-off values, which slightly changed their predictive values.

Surprisingly, we have not found any significant association between analysed frailty scores and the secondary outcomes: the duration of mechanical ventilation, the ICU, and hospital stay counted. Similarly, we did not find significant associations between the secondary outcomes and other baseline characteristics of patients, except for the age, STESS and serum troponin T, which were found to affect the length of hospital stay in our cohort.

All intensivists routinely perform biochemical tests at admission to intensive care. We can look at the obtained results from different angles. Standard biochemical parameter derangements may reflect the severity of acute critical illness. Moreover, they could originate from the long-term attenuation of homeostatic processes, though they are not considered in the definition of frailty. Finally, we must keep in mind that electrolyte disturbances, mainly hyponatremia, hypocalcemia, and hypomagnesemia, could worsen symptomatic seizures and significantly affect outcome scores. Therefore, early detection and careful correction of these biochemical or ionic disbalances are crucial to prevent permanent brain damage [12]. The predictive power of some biochemical markers tested by us is consistent with findings from similar studies. For example, the association between C-reactive protein and SE outcome, as identified by [25], influences in-hospital mortality and outcome at hospital discharge, however [26] showed less convincing associations. Despite the relatively well-known role of calcium in the pathophysiology of epilepsy and SE [27], there is still no conclusive association described between predictive powers of serum calcium or magnesium and SE outcomes, obviously attributed to limited specificity of standard serum ions in relation to SE.

We have not found any correlation between brain-specific serum S100 level at admission and measured outcomes, similarly as in [28]. However, we found the serum troponin T to be an independent risk factor for the outcome at hospital discharge in our cohort, indicating that this marker, though not of brain origin, could have some prognostic value in GOS assessment. We are not aware of any study analysing the link between the serum troponin T and the SE outcome, however, in one study the association between serum troponin I levels and SE was described [29]. There are no studies known to us, quantifying SE outcomes with frailty scores as analysed by us.

Specific new biomarkers that can be detected in blood and cerebrospinal fluid are increasingly investigated. As mentioned in the Introduction section, researchers have assessed the ability of biomarkers like neuron-specific enolase or progranulin to help diagnose SE [11, 28]. Above that, the neuron-specific enolase showed promising potential as a biomarker of EEG activity and seems to be helpful in assessing the risk of seizure recurrences [13]. A very recent review on fluid biomarkers described promising biomarkers of neuroglial injury which are increased in SE, namely, Neurofilaments (NfL), Ubiquitin C-terminal Hydrolase (UCH-L1), TAU and Phosphorylated-TAU (p-TAU), Glial Fibrillary Acid Protein (GFAC) or Vascular Endothelial Growth Factor (VEGF) [30]. Another two recent papers focused on the diagnostic and prognostic value of neutrophil to lymphocyte ratio in epilepsy and SE [31], and its association to the length of hospitalisation and the need for ICU admission [32]. Despite all the progress made, the prognostic value of many studied biomarkers still needs to be fully quantitatively resolved. We need further studies with extended follow-up periods to reliably evaluate their ability to predict outcomes of patients with SE admitted to intensive care units [11].

Our study has several limitations: (1) The study is not blinded since it is challenging to design a blinded study for frailty. (2) There are few missing biochemical readouts in some patients. (3) We enrolled not only the elderly but all adult patients as aging and physiological decline resulting in frailty is a continual process. Finally, it should be mentioned that many variables of no significant association with the tested outcomes would likely become significant if the number of enrolled individuals significantly increased. However, since this is a pilot study, we report all predictors that were found statistically significant in predicting the GOS. Among them, scores of frailty turn out to be very relevant.

## Conclusions

This pilot study identified promising prognostic powers of two frailty scores, the CFS and the mFI-11, comparable to age and STESS in predicting the GOS at hospital discharge, and three months later, in our cohort of comatose patients with SE. Only length of hospital stay was found to be associated with age, STESS, and serum troponin T regarding assessed secondary outcomes.

### Electronic supplementary material

Below is the link to the electronic supplementary material.

Supplementary Material 1

## Data Availability

The datasets generated and/or analyzed during the current study are not publicly available due to the anonymity of the participants. However, they are available from the corresponding author upon reasonable request.
