# Supplementary material for: Impact of frailty, biomarkers and basic biochemical parameters on outcomes of comatose patients in status epilepticus: a single-center prospective pilot study
Source: BMC Neurol. 2024 Jan 26;24:46. doi: 10.1186/s12883-024-03537-y (PMC10811840; doi:10.1186/s12883-024-03537-y)
Supplement: Supplementary file 1 — Supplementary Material 1 [file 12883_2024_3537_MOESM1_ESM.docx]

Supplementary information:

Suppl. Table 1

Descriptive statistics of measured variables.

| Parameter | unit | N | N (missing) | Mean | SD | p25 | p50 | p75 | min | max |
| --- | --- | --- | --- | --- | --- | --- | --- | --- | --- | --- |
| GOS_hosp |  | 30 | 0 | 3.60 | 1.35 | 3.00 | 4.00 | 5.00 | 1.00 | 5.00 |
| GOS 3M |  | 30 | 0 | 3.13 | 1.72 | 1.00 | 3.50 | 5.00 | 1.00 | 5.00 |
| MV_hours | hours | 30 | 0 | 132.33 | 160.99 | 22.50 | 51.50 | 202.00 | 0.00 | 523.00 |
| ICU_days | days | 30 | 0 | 7.83 | 8.28 | 2.00 | 5.00 | 10.75 | 1.00 | 32.00 |
| Hosp_days | days | 30 | 0 | 22.37 | 23.44 | 8.00 | 14.50 | 26.00 | 2.00 | 109.00 |
| Age | years | 30 | 0 | 56.53 | 15.90 | 44.00 | 56.00 | 67.75 | 28.00 | 86.00 |
| STESS |  | 30 | 0 | 3.27 | 1.26 | 2.00 | 3.00 | 4.00 | 2.00 | 6.00 |
| CFS |  | 30 | 0 | 3.43 | 1.79 | 2.00 | 3.00 | 4.00 | 1.00 | 8.00 |
| mFI-11 |  | 30 | 0 | 0.19 | 0.20 | 0.02 | 0.09 | 0.36 | 0.00 | 0.72 |
| GCS onset |  | 30 | 0 | 6.50 | 2.75 | 3.50 | 6.00 | 8.75 | 3.00 | 13.00 |
| Weight | kg | 30 | 0 | 77.27 | 18.63 | 63.25 | 75.00 | 86.75 | 46.00 | 130.00 |
| Height | cm | 30 | 0 | 168.57 | 7.78 | 163.50 | 169.50 | 175.00 | 151.00 | 185.00 |
| BMI | kg/m^2^ | 30 | 0 | 27.07 | 5.57 | 23.40 | 26.45 | 31.50 | 18.00 | 42.50 |
| Gly | mmol/l | 30 | 0 | 7.95 | 2.32 | 6.60 | 7.45 | 8.67 | 4.90 | 15.30 |
| S100 | µg/l | 30 | 11 | 0.26 | 0.28 | 0.10 | 0.14 | 0.26 | 0.05 | 1.20 |
| TNT | µmol/l | 30 | 6 | 61.79 | 92.43 | 9.25 | 20.50 | 55.50 | 3.00 | 339.00 |
| Na | mmol/l | 30 | 0 | 136.27 | 6.55 | 133.25 | 137.50 | 141.00 | 120.00 | 147.00 |
| K | mmol/l | 30 | 0 | 4.11 | 0.64 | 3.80 | 4.20 | 4.50 | 2.90 | 5.40 |
| Cl | mmol/l | 30 | 0 | 98.03 | 8.10 | 94.25 | 98.00 | 103.75 | 78.00 | 113.00 |
| P | mmol/l | 30 | 0 | 1.33 | 0.61 | 0.99 | 1.20 | 1.43 | 0.64 | 3.51 |
| Mg | mmol/l | 30 | 0 | 0.88 | 0.16 | 0.78 | 0.86 | 1.01 | 0.50 | 1.12 |
| Ca | mmol/l | 30 | 0 | 2.20 | 0.19 | 2.05 | 2.20 | 2.34 | 1.79 | 2.62 |
| CRP | mg/l | 30 | 0 | 20.64 | 42.25 | 1.42 | 5.15 | 18.30 | 0.10 | 216.80 |
| Alb | g/l | 30 | 2 | 34.57 | 4.88 | 31.12 | 33.10 | 37.30 | 25.40 | 46.30 |
| pH |  | 30 | 0 | 7.36 | 0.10 | 7.28 | 7.39 | 7.43 | 7.09 | 7.50 |
| pCO2 | kPa | 30 | 0 | 5.13 | 1.47 | 4.41 | 4.98 | 5.67 | 2.13 | 10.50 |
| BE | mmol/l | 30 | 0 | -3.65 | 6.12 | -6.15 | -3.20 | -0.03 | -25.00 | 10.00 |
| Lac | mmol/l | 30 | 2 | 2.85 | 2.37 | 1.12 | 2.00 | 3.38 | 0.70 | 10.50 |
| Sosm |  | 30 | 2 | 288.04 | 16.72 | 277.00 | 287.50 | 295.25 | 255.00 | 337.00 |
| OG |  | 30 | 7 | 3.43 | 10.87 | -3.50 | 1.00 | 6.00 | -6.00 | 44.00 |

N: number of patients; SD: standard deviation; GOS hosp: Glasgow Outcome Scale at hospital discharge; GCS 3M: Glasgow Outcome Scale three months after hospital discharge; MV: mechanical ventilation; ICU: intensive care unit; STESS: Status Epilepticus Severity Score at admission; CFS: Clinical Frailty Scale; mFI-11: Modified 11-item Frailty index; GCS: Glasgow Coma Scale; BMI: body mass index; S100: serum protein S-100; TNT: serum high-sensitivity Troponin T; Na: serum natrium; K: serum potassium; Cl: serum chloride; Mg: serum magnesium; P: serum phosphorus; Ca: serum calcium; Alb: serum albumin; CRP: C-reactive protein; Lac: blood lactate; pCO2: partial pressure of carbon dioxide; BE: base excess; Sosm: serum osmolality; OG: osmotic gap

Suppl. Table 2a

Parameters of univariate linear regression model for GOS hospital.

|  | Standardized Model | | | | Original Model | | | |
| --- | --- | --- | --- | --- | --- | --- | --- | --- |
| Characteristic | N | Beta | 95% CI | p-value | N | Beta | 95% CI | p-value |
| Age | 30 | -0.61 | -0.92, -0.31 | <0.001 | 30 | -0.05 | -0.08, -0.02 | 0.001 |
| Sex - Male |  | -0.64 | -1.4, 0.1 | 0.088 |  | -0.81 | -1.8, 0.20 | 0.11 |
| STESS | 30 | -0.47 | -0.81, -0.13 | 0.008 | 30 | -0.50 | -0.87, -0.13 | 0.010 |
| CFS | 30 | -0.70 | -0.98, -0.43 | <0.001 | 30 | -0.54 | -0.75, -0.34 | <0.001 |
| mFI_11 | 30 | -0.72 | -0.99, -0.45 | <0.001 | 30 | -4.7 | -6.7, -2.7 | <0.001 |
| BMI | 30 | -0.08 | -0.46, 0.31 | 0.7 | 30 | -0.01 | -0.10, 0.08 | 0.8 |
| GCS_onset | 30 | 0.30 | -0.07, 0.67 | 0.10 | 30 | 0.15 | -0.03, 0.33 | 0.10 |
| Alc_tox | 30 |  |  |  | 30 |  |  |  |
| Yes |  | 1.3 | -0.18, 2.7 | 0.084 |  | 1.5 | 0.48, 3.5 | 0.13 |
| Alc_ab | 30 | 30 |  |  |  |  |  |  |
| Yes |  | -0.40 | -1.2, 0.36 | 0.3 |  | -0.44 | -1.5, 0.59 | 0.4 |
| Nic_ab | 30 |  |  |  | 30 |  |  |  |
| Yes |  | 0.57 | -0.16, 1.3 | 0.12 |  | 0.67 | -0.33, 1.7 | 0.2 |
| Gly | 30 | -0.29 | -0.66, 0.08 | 0.12 | 30 | -0.20 | -0.41, 0.01 | 0.067 |
| Na | 30 | 0.07 | -0.32, 0.45 | 0.7 | 30 | 0.00 | -0.08, 0.08 | >0.9 |
| K | 30 | 0.26 | -0.12, 0.63 | 0.2 | 30 | 0.58 | -0.22, 1.4 | 0.15 |
| Cl | 30 | 0.10 | -0.29, 0.48 | 0.6 | 30 | 0.01 | -0.06, 0.07 | 0.8 |
| Mg | 30 | 0.27 | -0.10, 0.64 | 0.15 | 30 | 2.1 | -1.0, 5.2 | 0.2 |
| P | 30 | -0.01 | -0.40, 0.38 | >0.9 | 30 | 0.15 | -0.71, 1.0 | 0.7 |
| Ca | 30 | -0.39 | -0.75, -0.03 | 0.034 | 30 | -2.8 | -5.3, -0.34 | 0.028 |
| CRP | 30 | -0.15 | -0.53, 0.24 | 0.4 | 30 | 0.00 | -0.01, 0.01 | 0.7 |
| Alb | 28 | 0.09 | -0.32, 0.49 | 0.7 | 28 | 0.04 | -0.08, 0.15 | 0.5 |
| pH | 30 | -0.05 | -0.43, 0.34 | 0.8 | 30 | 0.42 | -4.8, 5.6 | 0.9 |
| pCO2 | 30 | -0.18 | -0.56, 0.20 | 0.3 | 30 | -0.20 | -0.54, 0.15 | 0.3 |
| BE | 30 | -0.10 | -0.48, 0.29 | 0.6 | 30 | -0.01 | -0.10, 0.07 | 0.7 |
| OG | 23 | 0.05 | -0.39, 0.49 | 0.8 | 23 | 0.02 | -0.03, 0.08 | 0.4 |
| TNT | 24 | -0.45 | -0.81, -0.10 | 0.014 | 24 | 0.00 | -0.01, 0.00 | 0.2 |
| S100 | 19 | 0.14 | -0.31, 0.58 | 0.5 | 19 | 0.71 | -1.2, 2.7 | 0.5 |
| Sosm | 28 | 0.29 | -0.08, 0.66 | 0.11 | 28 | 0.02 | -0.01, 0.05 | 0.2 |
| Lac | 28 | -0.22 | -0.62, 0.17 | 0.3 | 28 | -0.14 | -0.37, 0.09 | 0.2 |

N: number of patients ;CI: Confidence Interval; GOS hosp: Glasgow Outcome Scale at hospital discharge; GCS 3M: Glasgow Outcome Scale three months after hospital discharge; GCS: Glasgow Coma Scale; STESS: Status Epilepticus Severity Score at admission; CFS: Clinical Frailty Scale; mFI-11: Modified 11-item Frailty index; BMI: body mass index; Alc_ab: alcohol abuse; Alc_tox: alcohol intoxication; Nic_ab: nicotin abuse; S100: serum protein S-100; TNT: serum high-sensitivity Troponin T; Na: serum natrium; K: serum potassium; Cl: serum chloride; Mg: serum magnesium; P: serum phosphorus; Ca: serum calcium; Alb: serum albumin; CRP: C-reactive protein; Lac: lactate; pCO2: partial pressure of carbon dioxide; BE: base excess; Sosm: serum osmolality; OG: osmotic gap

Suppl. Table 2b

Parameters of univariate linear regression model for GOS three month.

|  | Standardized Model | | | | Original Model | | | |
| --- | --- | --- | --- | --- | --- | --- | --- | --- |
| Parameter | N | Beta | 95% CI | p-value | N | Beta | 95% CI | p-value |
| Age | 30 | -0.61 | -0.92, -0.31 | <0.001 | 30 | -0.06 | -0.10, -0.03 | <0.001 |
| Sex - Male | 18 | -0.40 | -1.2, 0.36 | 0.3 | 18 | -0.61 | -1.9, 0.70 | 0.3 |
| STESS | 30 | -0.46 | -0.80, -0.11 | 0.011 | 30 | -0.61 | -1.1, -0.14 | 0.013 |
| CFS | 30 | -0.65 | -0.94, -0.35 | <0.001 | 30 | -0.60 | -0.89, -0.31 | <0.001 |
| mFI_11 | 30 | -0.62 | -0.92, -0.32 | <0.001 | 30 | -5.2 | -7.9, -2.5 | <0.001 |
| BMI | 30 | 0.01 | -0.38, 0.40 | >0.9 | 30 | 0.01 | -0.11, 0.13 | >0.9 |
| GCS_onset | 30 | 0.22 | -0.15, 0.60 | 0.2 | 30 | 0.14 | -0.10, 0.37 | 0.2 |
| Alc_tox - Yes | 2 | 1.2 | -0.23, 2.7 | 0.10 | 2 | 2.0 | -0.50, 4.5 | 0.11 |
| Alc_ab - Yes | 12 | -0.39 | -1.2, 0.37 | 0.3 | 12 | -0.64 | -1.9, 0.67 | 0.3 |
| Nic_ab - Yes | 15 | 0.22 | -0.54, 0.97 | 0.6 | 15 | 0.27 | -1.0, 1.6 | 0.7 |
| Gly | 30 | -0.31 | -0.68, 0.06 | 0.10 | 30 | -0.23 | -0.50, 0.05 | 0.10 |
| Na | 30 | 0.16 | -0.22, 0.54 | 0.4 | 30 | 0.03 | -0.08, 0.13 | 0.6 |
| K | 30 | 0.21 | -0.17, 0.59 | 0.3 | 30 | 0.57 | -0.45, 1.6 | 0.3 |
| Cl | 30 | 0.19 | -0.19, 0.57 | 0.3 | 30 | 0.03 | -0.05, 0.11 | 0.4 |
| Mg | 30 | 0.38 | 0.02, 0.73 | 0.041 | 30 | 4.2 | 0.40, 8.0 | 0.032 |
| P | 30 | 0.00 | -0.38, 0.39 | >0.9 | 30 | 0.33 | -0.76, 1.4 | 0.5 |
| Ca | 30 | -0.43 | -0.78, -0.08 | 0.017 | 30 | -3.9 | -7.1, -0.84 | 0.015 |
| CRP | 30 | -0.21 | -0.59, 0.16 | 0.3 | 30 | -0.01 | -0.02, 0.01 | 0.3 |
| Alb | 28 | 0.10 | -0.29, 0.50 | 0.6 | 28 | 0.05 | -0.09, 0.19 | 0.5 |
| pH | 30 | -0.11 | -0.50, 0.27 | 0.6 | 30 | -1.5 | -8.1, 5.0 | 0.6 |
| pCO2 | 30 | -0.21 | -0.59, 0.16 | 0.3 | 30 | -0.31 | -0.74, 0.13 | 0.2 |
| BE | 30 | -0.21 | -0.58, 0.17 | 0.3 | 30 | -0.06 | -0.17, 0.05 | 0.3 |
| OG | 23 | 0.03 | -0.42, 0.48 | 0.9 | 23 | 0.03 | -0.04, 0.10 | 0.3 |
| TNT | 24 | -0.26 | -0.68, 0.15 | 0.2 | 24 | 0.00 | -0.01, 0.01 | 0.8 |
| S100 | 19 | 0.13 | -0.36, 0.62 | 0.6 | 19 | 0.91 | -2.0, 3.8 | 0.5 |
| Sosm | 28 | 0.33 | -0.05, 0.70 | 0.085 | 28 | 0.03 | 0.00, 0.07 | 0.084 |
| Lac | 28 | -0.11 | -0.51, 0.29 | 0.6 | 28 | -0.05 | -0.35, 0.24 | 0.7 |

N: number of patients; CI: Confidence Interval; GOS hosp: Glasgow Outcome Scale at hospital discharge; GCS 3M: Glasgow Outcome Scale three months after hospital discharge; GCS: Glasgow Coma Scale; STESS: Status Epilepticus Severity Score at admission; CFS: Clinical Frailty Scale; mFI-11: Modified 11-item Frailty index; BMI: body mass index; Alc_ab: alcohol abuse; Alc_tox: alcohol intoxication; Nic_ab: nicotine abuse; S100: serum protein S-100; TNT: serum high-sensitivity Troponin T; Na: serum natrium; K: serum potassium; Cl: serum chloride; Mg: serum magnesium; P: serum phosphorus; Ca: serum calcium; Alb: serum albumin; CRP: C-reactive protein; Lac: lactate; pCO2: partial pressure of carbon dioxide; BE: base excess; Sosm: serum osmolality; OG: osmotic gap

Suppl. Table 3

Comparison of parameters of logistic regression model for hospital and three month GOS.

| Logistic regression on binarized data (untransformed data) | | | | | | | | | | |
| --- | --- | --- | --- | --- | --- | --- | --- | --- | --- | --- |
| GOS hospital | | | | |  | GOS 3 month | | | | |
| parameter | N | OR | 95% CI | p-value |  | parameter | N | OR | 95% CI | p-value |
| CFS_3 | 30 |  |  |  |  | CFS_3 | 30 |  |  |  |
| >3 |  | — | — |  |  | >3 |  | — | — |  |
| <=3 |  | 7.467 | 1.524, 46.35 | 0.019 |  | <=3 |  | 9.750 | 1.838, 78.61 | 0.014 |
| mFI-11 09 | 30 |  |  |  |  | mFI-11 09 | 30 |  |  |  |
| >0.09 |  | — | — |  |  | >0.09 |  | — | — |  |
| <=0.09 |  | 5.200 | 1.132, 28.09 | 0.041 |  | <=0.09 |  | 6.000 | 1.271, 35.65 | 0.032 |
| Age 56 | 30 |  |  |  |  | Age_52 | 30 |  |  |  |
| >56 |  | — | — |  |  | >52 |  | — | — |  |
| <=56 |  | 10.83 | 2.179, 70.73 | 0.006 |  | <=52 |  | 13.00 | 2.414, 107.4 | 0.006 |
| STESS_3 | 30 |  |  |  |  | STESS_3 | 30 |  |  |  |
| >3 |  | — | — |  |  | >3 |  | — | — |  |
| <=3 |  | 7.467 | 1.524, 46.35 | 0.019 |  | <=3 |  | 9.750 | 1.838, 78.61 | 0.014 |
| Ca_2.04 | 30 |  |  |  |  | Ca_2.17 | 30 |  |  |  |
| >2.04 |  | — | — |  |  | >2.17 |  | — | — |  |
| <=2.04 |  | 6.545 | 0.915, 134.2 | 0.10 |  | <=2.17 |  | 3.000 | 0.699, 14.26 | 0.15 |
|  |  |  |  |  |  | Mg_0.85 | 30 |  |  |  |
|  |  |  |  |  |  | <=0.85 |  | — | — |  |
|  |  |  |  |  |  | >0.85 |  | 4.000 | 0.918, 19.85 | 0.074 |
| TNT_41 | 24 |  |  |  |  | TNT_7 | 24 |  |  |  |
| >41 |  | — | — |  |  | >=7 |  | — | — |  |
| <=41 |  | 14.40 | 1.830, 312.2 | 0.027 |  | <7 |  | 6.857 | 0.813, 149.4 | 0.11 |
| GCS_onset_8 | 30 |  |  |  |  | GCS_onset_7.5 | 30 |  |  |  |
| <=8 |  | — | — |  |  | <=7.5 |  | — | — |  |
| >8 |  | 3.000 | 0.549, 23.69 | 0.2 |  | >7.5 |  | 3.500 | 0.735, 20.30 | 0.13 |
| CRP_3.1 | 30 |  |  |  |  | CRP_2.3 | 30 |  |  |  |
| >3.1 |  | — | — |  |  | >2.3 |  | — | — |  |
| <=3.1 |  | 22.00 | 3.181, 453.7 | 0.008 |  | <=2.3 |  | 28.00 | 3.944, 586.8 | 0.004 |
| Sosm_298 | 28 |  |  |  |  | Sosm_298 | 28 |  |  |  |
| <298 |  | — | — |  |  | <298 |  | — | — |  |
| >=298 |  | — | 0.000, NA | >0.9 |  | >=298 |  | — | 0.000, NA | >0.9 |
| Gly_6 | 30 |  |  |  |  | Gly_6 | 30 |  |  |  |
| >=6 |  | — | — |  |  | >=6 |  | — | — |  |
| <6 |  | — | 0.000, NA | >0.9 |  | <6 |  | — | 0.000, NA | >0.9 |
| pCO2_5.25 | 30 |  |  |  |  | pCO2_4.5 | 30 |  |  |  |
| <=5.25 |  | — | — |  |  | >4.5 |  | — | — |  |
| >5.25 |  | 2.963 | 0.634, 16.90 | 0.2 |  | <=4.5 |  | 2.667 | 0.546, 15.52 | 0.2 |
| K_4.3 | 30 |  |  |  |  | K_4.15 | 30 |  |  |  |
| <4.3 |  | — | — |  |  | <4.15 |  | — | — |  |
| >=4.3 |  | 2.531 | 0.577, 12.52 | 0.2 |  | >=4.15 |  | 1.714 | 0.406, 7.592 |  |
| BE_0.3 | 30 |  |  |  |  | BE_3.75 | 30 |  |  |  |
| <0.3 |  | — | — |  |  | <3.75 |  | — | — |  |
| >=0.3 |  | 2.292 | 0.400, 18.38 | 0.4 |  | >=3.75 |  | — | 0.000, NA | >0.9 |
| Cl_104 | 30 |  |  |  |  | Cl_102.5 | 30 |  |  |  |
| <104 |  | — | — |  |  | <102.5 |  | — | — |  |
| >=104 |  | 3.000 | 0.549, 23.69 | 0.2 |  | >=102.5 |  | 3.500 | 0.735, 20.30 | 0.13 |
| Na_137 | 30 |  |  |  |  | Na_137 | 30 |  |  |  |
| <137 |  | — | — |  |  | <137 |  | — | — |  |
| >=137 |  | 2.800 | 0.634, 13.50 | 0.2 |  | >=137 |  | 3.143 | 0.709, 15.83 | 0.14 |
| OG_9 | 23 |  |  |  |  | OG_8 | 23 |  |  |  |
| <9 |  | — | — |  |  | <8 |  | — | — |  |
| >=9 |  | — | 0.000, NA | >0.9 |  | >=8 |  | 6.286 | 0.739, 137.4 | 0.13 |
| pH_7.409 | 30 |  |  |  |  | pH_7.375 | 30 |  |  |  |
| >=7.409 |  | — | — |  |  | >=7.375 |  | — | — |  |
| <7.409 |  | 2.139 | 0.495, 9.823 | 0.3 |  | <7.375 |  | 3.000 | 0.699, 14.26 | 0.15 |
| Lactate_2.1 | 28 |  |  |  |  | Lac_1.9 | 28 |  |  |  |
| >2.1 |  | — | — |  |  | >1.9 |  | — | — |  |
| <=2.1 |  | 1.750 | 0.392, 8.207 | 0.5 |  | <=1.9 |  | 2.400 | 0.537, 11.70 | 0.3 |
| S100_0.191 | 19 |  |  |  |  | S100_0.191 | 19 |  |  |  |
| <0.191 |  | — | — |  |  | <0.191 |  | — | — |  |
| >=0.191 |  | 2.500 | 0.366, 22.74 | 0.4 |  | >=0.191 |  | 3.600 | 0.538, 33.07 | 0.2 |
| Alb_29 | 28 |  |  |  |  | Alb_32 | 28 |  |  |  |
| <=29 |  | — | — |  |  | <32 |  | — | — |  |
| >29 |  | 0.000 | >0.9 |  |  | >=32 |  | 2.750 | 0.549, 16.38 | 0.2 |
| P_1.1 | 30 |  |  |  |  | P_1.27 | 30 |  |  |  |
| >1.1 |  | — | — |  |  | >1.27 |  | — | — |  |
| <=1.1 |  | 3.214 | 0.732, 16.12 | 0.13 |  | <=1.27 |  | 2.286 | 0.533, 10.61 | 0.3 |
| BMI_22.8 | 30 |  |  |  |  | BMI_30 | 30 |  |  |  |
| >22.8 |  | — | — |  |  | <30 |  | — | — |  |
| <=22.8 |  | 2.292 | 0.400, 18.38 | 0.4 |  | >=30 |  | 1.833 | 0.398, 9.183 | 0.4 |
| Sex | 30 |  |  |  |  | Sex | 30 |  |  |  |
| Male |  | — | — |  |  | Male |  | — | — |  |
| Female |  | 3.750 | 0.809, 21.49 | 0.11 |  | Female |  | 3.143 | 0.709, 15.83 | 0.14 |
| Alc_tox | 30 |  |  |  |  | Alc_tox | 30 |  |  |  |
| No |  | — | — |  |  | No |  | — | — |  |
| Yes |  | — | 0.000, NA | >0.9 |  | Yes |  | — | 0.000, NA | >0.9 |
| Alc_ab | 30 |  |  |  |  | Alc_ab | 30 |  |  |  |
| Yes |  | — | — |  |  | Yes |  | — | — |  |
| No |  | 1.571 | 0.357, 7.102 | 0.5 |  | No |  | 1.750 | 0.403, 8.048 | 0.5 |
| Nic_ab | 30 |  |  |  |  | Nic_ab | 30 |  |  |  |
| No |  | — | — |  |  | No |  | — | — |  |
| Yes |  | 4.125 | 0.930, 21.22 | 0.072 |  | Yes |  | 2.250 | 0.532, 10.22 | 0.3 |

Comparison of logistic regression models of binarized explanatory variables predicting GOS hosp and GOS 3M. Their prediction strengths, measured by OR, its CI, and p-values regarding GOS hosp and GOS 3M could be compared.

N: number of patients; CI: Confidence Interval; OR: odds ratio; GOS: Glasgow Outcome Scale; GCS: Glasgow Coma Scale onset: STESS: Status Epilepticus Severity Score at admission; CFS: Clinical Frailty Scale; mFI-11: Modified 11-item Frailty index; BMI: body mass index; Alc_ab: alcohol abuse; Alc_tox: alcohol intoxication; Nic_ab: nicotine abuse; S100: serum protein S-100; TNT: serum high-sensitivity Troponin T; Na: serum natrium; K: serum potassium; Cl: serum chloride; Mg: serum magnesium; P: serum phosphorus; Ca: serum calcium; Alb: serum albumin; CRP: C-reactive protein; Lac: lactate; pCO2: partial pressure of carbon dioxide; BE: base excess; Sosm: serum osmolality; OG: osmotic gap

Suppl. Table 4.

Explanatory data analysis of evaluated parameters.

| Exploratory data analysis on binarized exploratory parameters | | | | | | | | | | |
| --- | --- | --- | --- | --- | --- | --- | --- | --- | --- | --- |
|  |  | GOS hosp | |  |  |  |  | GOS 3M | |  |
| Parameter | N | 1-3, N=13 | 4-5, N=17 | p-value |  | Parameter | N | 1-3, N=15 | 4-5, N=15 | p-value |
|  |  |  |  |  |  |  |  |  |  |  |
| CFS 3 | 30 |  |  | 0.023 |  | CFS3 | 30 |  |  | 0.008 |
| >3 |  | 8.0 (61.5%) | 3.0 (17.6%) |  |  | >3 |  | 9.0 (60.0%) | 2.0 (13.3%) |  |
| <=3 |  | 5.0 (38.5%) | 14.0 (82.4%) |  |  | <=3 |  | 6.0 (40.0%) | 13.0 (86.7%) |  |
| mFI11 09 | 30 |  |  | 0.035 |  | mFI11 09 | 30 |  |  | 0.025 |
| >0.09 |  | 8.0 (61.5%) | 4.0 (23.5%) |  |  | >0.09 |  | 9.0 (60.0%) | 3.0 (20.0%) |  |
| <=0.09 |  | 5.0 (38.5%) | 13.0 (76.5%) |  |  | <=0.09 |  | 6.0 (40.0%) | 12.0 (80.0%) |  |
| Age 56 | 30 |  |  | 0.004 |  | Age 52 | 30 |  |  | 0.003 |
| >56 |  | 10.0 (76.9%) | 4.0 (23.5%) |  |  | >52 |  | 13.0 (86.7%) | 5.0 (33.3%) |  |
| <=56 |  | 3.0 (23.1%) | 13.0 (76.5%) |  |  | <=52 |  | 2.0 (13.3%) | 10.0 (66.7%) |  |
| STESS 3 | 30 |  |  | 0.023 |  | STESS 3 | 30 |  |  | 0.008 |
| >3 |  | 8.0 (61.5%) | 3.0 (17.6%) |  |  | >3 |  | 9.0 (60.0%) | 2.0 (13.3%) |  |
| <=3 |  | 5.0 (38.5%) | 14.0 (82.4%) |  |  | <=3 |  | 6.0 (40.0%) | 13.0 (86.7%) |  |
| Ca 2.04 | 30 |  |  | 0.10 |  | Ca 2.17 | 30 |  |  | 0.14 |
| >2.04 |  | 12.0 (92.3%) | 11.0 (64.7%) |  |  | >2.17 |  | 10.0 (66.7%) | 6.0 (40.0%) |  |
| <=2.04 |  | 1.0 (7.7%) | 6.0 (35.3%) |  |  | <=2.17 |  | 5.0 (33.3%) | 9.0 (60.0%) |  |
| TNT 41 | 24 |  |  | 0.023 |  | TNT 7 | 24 |  |  | 0.14 |
| >41 |  | 6.0 (54.5%) | 1.0 (7.7%) |  |  | >=7 |  | 12.0 (92.3%) | 7.0 (63.6%) |  |
| <=41 |  | 5.0 (45.5%) | 12.0 (92.3%) |  |  | <7 |  | 1.0 (7.7%) | 4.0 (36.4%) |  |
| Unknown |  | 2 | 4 |  |  | Unknown |  | 2 | 4 |  |
| GCS onset 8 | 30 |  |  | 0.4 |  | GCS onset 7.5 | 30 |  |  | 0.12 |
| <=8 |  | 11.0 (84.6%) | 11.0 (64.7%) |  |  | <=7.5 |  | 12.0 (80.0%) | 8.0 (53.3%) |  |
| >8 |  | 2.0 (15.4%) | 6.0 (35.3%) |  |  | >7.5 |  | 3.0 (20.0%) | 7.0 (46.7%) |  |
| CRP 3.1 | 30 |  |  | 0.002 |  | CRP 2.3 | 30 |  |  | <0.001 |
| >3.1 |  | 12.0 (92.3%) | 6.0 (35.3%) |  |  | >2.3 |  | 14.0 (93.3%) | 5.0 (33.3%) |  |
| <=3.1 |  | 1.0 (7.7%) | 11.0 (64.7%) |  |  | <=2.3 |  | 1.0 (6.7%) | 10.0 (66.7%) |  |
| Sosm 298 | 28 |  |  | 0.053 |  | Sosm298 | 28 |  |  | 0.041 |
| <298 |  | 12.0 (100.0%) | 11.0 (68.8%) |  |  | <298 |  | 14.0 (100.0%) | 9.0 (64.3%) |  |
| >=298 |  | 0.0 (0.0%) | 5.0 (31.3%) |  |  | >=298 |  | 0.0 (0.0%) | 5.0 (35.7%) |  |
| Unknown |  | 1 | 1 |  |  | Unknown |  | 1 | 1 |  |
| Gly 6 | 30 |  |  | 0.052 |  | Gly 6 | 30 |  |  | 0.042 |
| >=6 |  | 13.0 (100.0%) | 12.0 (70.6%) |  |  | >=6 |  | 15.0 (100.0%) | 10.0 (66.7%) |  |
| <6 |  | 0.0 (0.0%) | 5.0 (29.4%) |  |  | <6 |  | 0.0 (0.0%) | 5.0 (33.3%) |  |
| pCO2 5.25 | 30 |  |  | 0.3 |  | pCO2 4.5 | 30 |  |  | 0.4 |
| <=5.25 |  | 10.0 (76.9%) | 9.0 (52.9%) |  |  | >4.5 |  | 12.0 (80.0%) | 9.0 (60.0%) |  |
| >5.25 |  | 3.0 (23.1%) | 8.0 (47.1%) |  |  | <=4.5 |  | 3.0 (20.0%) | 6.0 (40.0%) |  |
| K 4.3 | 30 |  |  | 0.2 |  | K 4.15 | 30 |  |  | 0.5 |
| <4.3 |  | 9.0 (69.2%) | 8.0 (47.1%) |  |  | <4.15 |  | 8.0 (53.3%) | 6.0 (40.0%) |  |
| >=4.3 |  | 4.0 (30.8%) | 9.0 (52.9%) |  |  | >=4.15 |  | 7.0 (46.7%) | 9.0 (60.0%) |  |
| BE 0.3 | 30 |  |  | 0.4 |  | BE 3.75 | 30 |  |  | >0.9 |
| <0.3 |  | 11.0 (84.6%) | 12.0 (70.6%) |  |  | <3.75 |  | 15.0 (100.0%) | 14.0 (93.3%) |  |
| >=0.3 |  | 2.0 (15.4%) | 5.0 (29.4%) |  |  | >=3.75 |  | 0.0 (0.0%) | 1.0 (6.7%) |  |
| Cl 104 | 30 |  |  | 0.4 |  | Cl 102.5 | 30 |  |  | 0.12 |
| <104 |  | 11.0 (84.6%) | 11.0 (64.7%) |  |  | <102.5 |  | 12.0 (80.0%) | 8.0 (53.3%) |  |
| >=104 |  | 2.0 (15.4%) | 6.0 (35.3%) |  |  | >=102.5 |  | 3.0 (20.0%) | 7.0 (46.7%) |  |
| Na 137 | 30 |  |  | 0.2 |  | Na 137 | 30 |  |  | 0.14 |
| <137 |  | 7.0 (53.8%) | 5.0 (29.4%) |  |  | <137 |  | 8.0 (53.3%) | 4.0 (26.7%) |  |
| >=137 |  | 6.0 (46.2%) | 12.0 (70.6%) |  |  | >=137 |  | 7.0 (46.7%) | 11.0 (73.3%) |  |
| OG 9 | 23 |  |  | 0.2 |  | OG 8 | 23 |  |  | 0.2 |
| <9 |  | 11.0 (100.0%) | 9.0 (75.0%) |  |  | <8 |  | 11.0 (91.7%) | 7.0 (63.6%) |  |
| >=9 |  | 0.0 (0.0%) | 3.0 (25.0%) |  |  | >=8 |  | 1.0 (8.3%) | 4.0 (36.4%) |  |
| Unknown |  | 2 | 5 |  |  | Unknown |  | 3 | 4 |  |
| pH 7.409 | 30 |  |  | 0.3 |  | pH 7.375 | 30 |  |  | 0.14 |
| >=7.409 |  | 7.0 (53.8%) | 6.0 (35.3%) |  |  | >=7.375 |  | 10.0 (66.7%) | 6.0 (40.0%) |  |
| <7.409 |  | 6.0 (46.2%) | 11.0 (64.7%) |  |  | <7.375 |  | 5.0 (33.3%) | 9.0 (60.0%) |  |
| Lac 2.1 | 28 |  |  | 0.5 |  | Lac 1.9 | 28 |  |  | 0.3 |
| >2.1 |  | 7.0 (53.8%) | 6.0 (40.0%) |  |  | >1.9 |  | 9.0 (60.0%) | 5.0 (38.5%) |  |
| <=2.1 |  | 6.0 (46.2%) | 9.0 (60.0%) |  |  | <=1.9 |  | 6.0 (40.0%) | 8.0 (61.5%) |  |
| Unknown |  | 0 | 2 |  |  | Unknown |  | 0 | 2 |  |
| S100 0.191 | 19 |  |  | 0.6 |  | S100 0.191 | 19 |  |  | 0.4 |
| <0.191 |  | 5.0 (71.4%) | 6.0 (50.0%) |  |  | <0.191 |  | 6.0 (75.0%) | 5.0 (45.5%) |  |
| >=0.191 |  | 2.0 (28.6%) | 6.0 (50.0%) |  |  | >=0.191 |  | 2.0 (25.0%) | 6.0 (54.5%) |  |
| Unknown |  | 6 | 5 |  |  | Unknown |  | 7 | 4 |  |
| Alb 29 | 28 |  |  | 0.2 |  | Alb 32 | 28 |  |  | 0.4 |
| <=29 |  | 0.0 (0.0%) | 3.0 (20.0%) |  |  | <32 |  | 6.0 (42.9%) | 3.0 (21.4%) |  |
| >29 |  | 13.0 (100.0%) | 12.0 (80.0%) |  |  | >=32 |  | 8.0 (57.1%) | 11.0 (78.6%) |  |
| Unknown |  | 0 | 2 |  |  | Unknown |  | 1 | 1 |  |
| BMI 22.8 | 30 |  |  | 0.4 |  | BMI 30 | 30 |  |  | 0.4 |
| >22.8 |  | 11.0 (84.6%) | 12.0 (70.6%) |  |  | <30 |  | 11.0 (73.3%) | 9.0 (60.0%) |  |
| <=22.8 |  | 2.0 (15.4%) | 5.0 (29.4%) |  |  | >=30 |  | 4.0 (26.7%) | 6.0 (40.0%) |  |
| P 1.1 | 30 |  |  | 0.13 |  | P 1.27 | 30 |  |  | 0.3 |
| >1.1 |  | 9.0 (69.2%) | 7.0 (41.2%) |  |  | >1.27 |  | 8.0 (53.3%) | 5.0 (33.3%) |  |
| <=1.1 |  | 4.0 (30.8%) | 10.0 (58.8%) |  |  | <=1.27 |  | 7.0 (46.7%) | 10.0 (66.7%) |  |
| Sex | 30 |  |  | 0.10 |  | Sex | 30 |  |  | 0.14 |
| Male |  | 10.0 (76.9%) | 8.0 (47.1%) |  |  | Male |  | 11.0 (73.3%) | 7.0 (46.7%) |  |
| Female |  | 3.0 (23.1%) | 9.0 (52.9%) |  |  | Female |  | 4.0 (26.7%) | 8.0 (53.3%) |  |
| Alc_tox | 30 | 0.0 (0.0%) | 2.0 (11.8%) | 0.5 |  | Alc_tox | 30 |  |  | 0.5 |
|  |  |  |  |  |  | No |  | 15.0 (100.0%) | 13.0 (86.7%) |  |
|  |  |  |  |  |  | Yes |  | 0.0 (0.0%) | 2.0 (13.3%) |  |
| Alc_ab | 30 | 6.0 (46.2%) | 6.0 (35.3%) | 0.5 |  | Alc_ab | 30 |  |  | 0.5 |
|  |  |  |  |  |  | Yes |  | 7.0 (46.7%) | 5.0 (33.3%) |  |
|  |  |  |  |  |  | No |  | 8.0 (53.3%) | 10.0 (66.7%) |  |
| Nic_ab | 30 | 4.0 (30.8%) | 11.0 (64.7%) | 0.065 |  | Nic_ab | 30 |  |  | 0.3 |
|  |  |  |  |  |  | No |  | 9.0 (60.0%) | 6.0 (40.0%) |  |
|  |  |  |  |  |  | Yes |  | 6.0 (40.0%) | 9.0 (60.0%) |  |

The table compares the ability of binarized (predictor parameter is <, or > than its cut-off value estimated by maximization of Youden index) explanatory variables to predict GOS 1-3 and GOS 4-5, evaluated by Pearson’s Chi-squared test and Fisher’s exact test. Explanatory variables with *p*-values smaller than 0.05 were considered statistically significant in predicting GOS.
N: number of patients; GOS hosp: Glasgow Outcome Scale at hospital discharge; GCS 3M: Glasgow Outcome Scale three months after hospital discharge; GCS: Glasgow Coma Scale; STESS: Status Epilepticus Severity Score at admission; CFS: Clinical Frailty Scale; mFI-11: Modified 11-item Frailty index; BMI: body mass index; Alc_ab: alcohol abuse; Alc_tox: alcohol intoxication; Nic_ab: nicotine abuse; S100: serum protein S-100; TNT: serum high-sensitivity Troponin T; Na: serum natrium; K: serum potassium; Cl: serum chloride; Mg: serum magnesium; P: serum phosphorus; Ca: serum calcium; Alb: serum albumin; CRP: C-reactive protein; Lac: lactate; pCO2: partial pressure of carbon dioxide; BE: base excess; Sosm: serum osmolality; OG: osmotic gap

Suppl. Figure 1

Boxplots illustrating the distributions of dependent and independent variables.

GOS hosp: Glasgow Outcome Scale at hospital discharge; GCS 3M: Glasgow Outcome Scale three months after hospital discharge; GCS: Glasgow Coma Scale; STESS: Status Epilepticus Severity Score at admission; CFS: Clinical Frailty Scale; mFI-11: Modified 11-item Frailty index; BMI: body mass index; Alc_ab: alcohol abuse; Alc_tox: alcohol intoxication; Nic_ab: nicotine abuse; S100: serum protein S-100; TNT: serum high-sensitivity Troponin T; Na: serum natrium; K: serum potassium; Cl: serum chloride; Mg: serum magnesium; P: serum phosphorus; Ca: serum calcium; Alb: serum albumin; CRP: C-reactive protein; Lac: arterial blood lactate; pCO2: partial pressure of carbon dioxide; BE: base excess; Sosm: serum osmolality; OG: osmotic gap.

Suppl. Figure 2:

Pearson correlation plot with colormap indicating the strength of correlations in our datasets.

GOS hosp: Glasgow Outcome Scale at hospital discharge; GCS 3M: Glasgow Outcome Scale three months after hospital discharge; GCS: Glasgow Coma Scale; STESS: Status Epilepticus Severity Score at admission; CFS: Clinical Frailty Scale; mFI-11: Modified 11-item Frailty index; BMI: body mass index; Alc_ab: alcohol abuse; Alc_tox: alcohol intoxication; Nic_ab: nicotine abuse; S100: serum protein S-100; TNT: serum high-sensitivity Troponin T; Na: serum natrium; K: serum potassium; Cl: serum chloride; Mg: serum magnesium; P: serum phosphorus; Ca: serum calcium; Alb: serum albumin; CRP: C-reactive protein; Lac: lactate; pCO2: partial pressure of carbon dioxide; BE: arterial blood base excess; Sosm: serum osmolality; OG: osmotic gap.

Suppl. Figure 3

Graphical summarisation of exploratory data analysis showing variables of the highest identified strength regarding the GOS prediction.


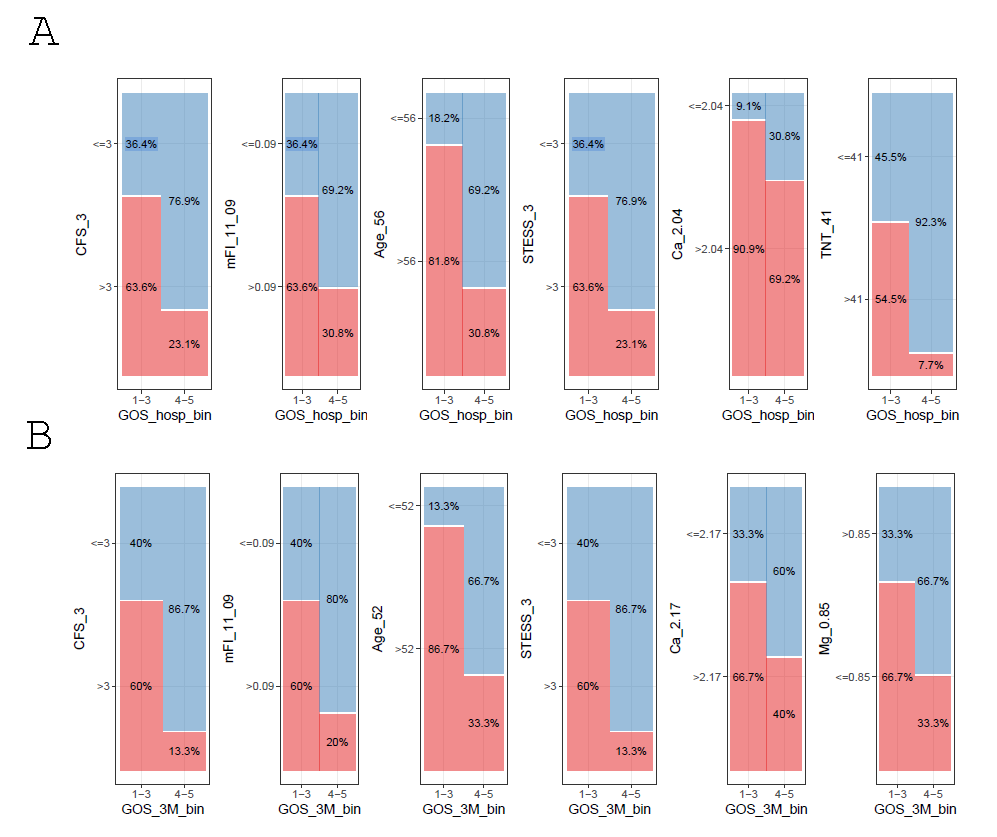


The more significant the difference in heights of red columns in each graph, the stronger the GOS predicting power of corresponding binarized variables.

GOS hosp: Glasgow Outcome Scale at hospital discharge; GCS 3M: Glasgow Outcome Scale three months after hospital discharge; STESS: Status Epilepticus Severity Score at admission; CFS: Clinical Frailty Scale; mFI-11: Modified 11-item Frailty index; Ca: serum calcium; Mg: serum magnesium; TNT: serum high-sensitivity Troponin T.

Figure 4

Receiver operating characteristic (ROC) curves of the most relevant predictors identified in our datasets (binarized untransformed data).


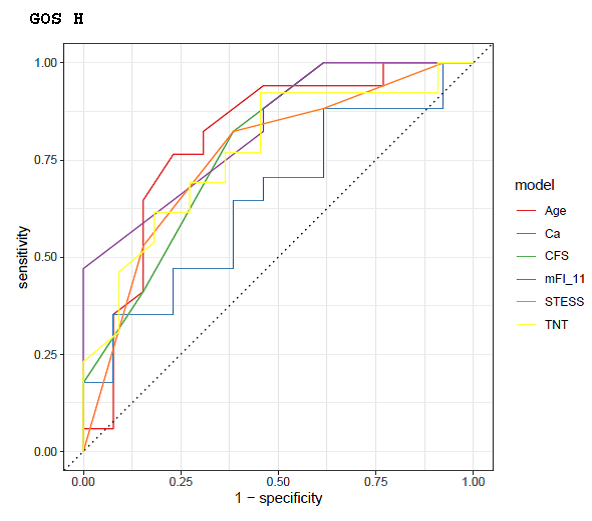

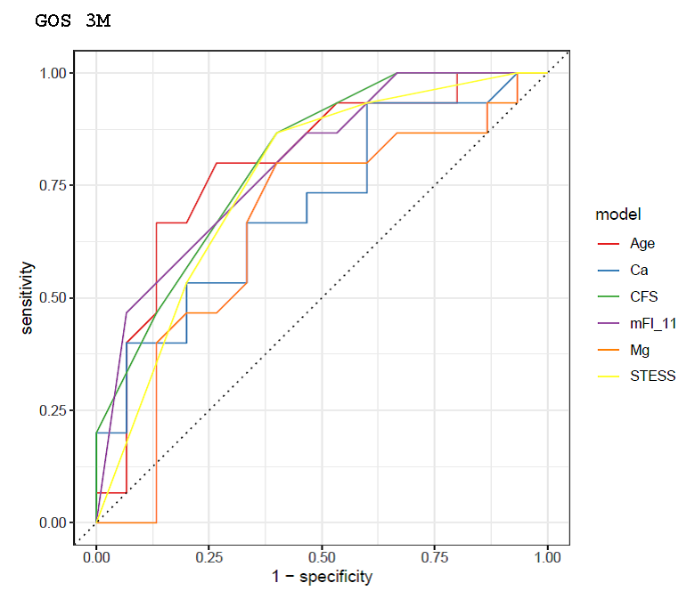


These ROC curves characterize how changes in a given explanatory parameter affect the prediction of the GOS logistic regression estimator as discrimination thresholds vary.

GOS hosp: Glasgow Outcome Scale at hospital discharge; GOS 3M: Glasgow Outcome Scale three months after hospital discharge; STESS: Status Epilepticus Severity Score at admission; CFS: Clinical Frailty Scale; mFI-11: Modified 11-item Frailty index; Ca: serum calcium; Mg: serum magnesium; TNT: serum high-sensitivity Troponin T.
